# Experimental evidence of auxeticity in ion implanted single crystal calcite

**DOI:** 10.1038/s41598-022-10177-0

**Published:** 2022-04-12

**Authors:** Michael E. Liao, Chao Li, Nachiket Shah, Yi-Hsuan Hsiao, Mathieu Bauchy, Gaurav Sant, Mark S. Goorsky

**Affiliations:** 1 Materials Science and Engineering, University of California, Los Angeles, Los Angeles, CA 90095 USA; 2 Applied Materials, Santa Clara, CA 95054 USA; 3 Materials Science and Engineering, University of Illinois Urbana-Champaign, Urbana, IL 61801 USA; 4 Civil and Environmental Engineering, University of California, Los Angeles, Los Angeles, CA 90095 USA

**Keywords:** Materials science, Structural materials, Civil engineering

## Abstract

We report initial experimental evidence of auxeticity in calcite by ion implanting (1010) oriented single crystalline calcite with Ar^+^ at room temperature using an ion energy of 400 keV and a dose of 1 × 10^14^ cm^−2^. Lattice compression normal to the substrate surface was observed, which is an atypical result for ion implanted materials. The auxetic behavior is consistent with predictions that indicate auxeticity had been predicted along two crystallographic directions including [1010]. Materials with a positive Poisson’s ratio experience lattice expansion normal to the substrate surface when ion implanted, whereas lattice contraction normal to the surface is evidence of auxetic behavior. Triple-axis X-ray diffraction measurements confirmed the auxetic strain state of the implanted calcite substrates. Reciprocal space maps for the symmetric 3030 and asymmetric 1450 reflections revealed that the implanted region was fully strained (pseudomorphic) to the bulk of the substrate, as is typical with implanted single crystals. A symmetric (3030) ω:2θ line scan was used with X-ray dynamical diffraction simulations to model the strain profile and extract the variation of compressive strain as a function of depth normal to the substrate surface. SRIM calculations were performed to obtain a displacement-per-atom profile and implanted Ar^+^ concentration profile. It was found that the strain profile matches the displacement-per-atom profile. This study demonstrated the use of ion implantation and X-ray diffraction methods to probe mechanical properties of materials and to test predictions such as the auxeticity.

## Introduction

Auxeticity—material with a negative Poisson’s ratio—is a characteristic that has been recognized for enhanced mechanical properties and is often associated with foams and metamaterials^1–3^. Crystalline materials make up a smaller fraction of known materials that exhibit auxeticity^3,4^. Calcite, a polymorph of calcium carbonate with a trigonal crystal structure^5^, has been predicted to exhibit auxetic behavior^6^ but this has not yet been experimentally observed. Calculations by Aouni et al.^6^ indicate that calcite should exhibit auxeticity along two crystallographic directions: [1010] and a high index direction 48° tilted from the [0001] with an in-plane component of [1230]. The expected Poisson ratio along the [1010] is − 0.0249^6^, but no discussion on the mechanism of auxeticity was given. Hence, one approach to test this prediction for calcite is to measure its strain behavior upon ion implantation. In this process, ions are accelerated towards a target material and penetrate the material ranging from nanometers to microns depending on the implantation parameters used, such as the implant species energy (eV to MeV), incident angle, and species^7^. These implanted ions knock target atoms off the lattice sites and these displaced species elastically distort the lattice. While the implanted ions induce lattice distortion in the implanted region of the material, the underlying (and much thicker) substrate material maintains its unstrained state and thus imposes in-plane biaxial stress, producing a pseudomorphic strain state in the implanted “layer.” A material with a positive Poisson’s ratio would exhibit out-of-plane expansion in response to the in-plane compressive biaxial stress from the substrate. In contrast, an implanted auxetic material would be expected to exhibit out-of-plane lattice compression in response to the in-plane biaxial compressive stress from the substrate. Early work by Servidori^8^ for ion implanted silicon substrates showed that this distortion can be studied with X-ray diffraction (XRD) and a strain profile caused by the ion implantation can be extracted. Previous works have examined a wide roster of materials including Si^9^, III–V^10,11^, and II–VI^12^ materials and employ both XRD measurements and Monte Carlo simulations to obtain strain and displacements-per-atom profiles of implanted materials. Out-of-plane lattice expansion due to the implantation is universally observed for previously studied materials. Previous work examined the effect of Ar^+^ implanted (1010) calcite substrates^13,14^ but the implant-induced strain was not measured. While the reported molecular dynamics simulations suggest implant-induced lattice expansion^13^ of Ar^+^ implanted calcite, these calculations neglect the in-plane compressive biaxial stress from the underlying substrate. The Ar^+^ energy used in those earlier studies—and here—was 400 keV, which corresponds to a projected range of ~ 400 nm from the substrate surface (with a substrate thickness of 1 mm).

In this work, auxeticity in calcite is experimentally determined by measuring both the out-of-plane and in-plane strain state of Ar^+^ implanted single crystalline (1010) calcite using triple-axis X-ray diffraction measurements. The magnitude and type of strain (compressive vs tensile) is observed by the position of the strained layer reflection in reference to the substrate reflection. The strain distribution is quantified through dynamical diffraction modeling of the X-ray diffraction scans. We demonstrate that using ion implantation and strain analysis via X-ray diffraction is a suitable approach for probing the mechanical properties of crystalline materials.

## Results and discussion

**Figure 1:**
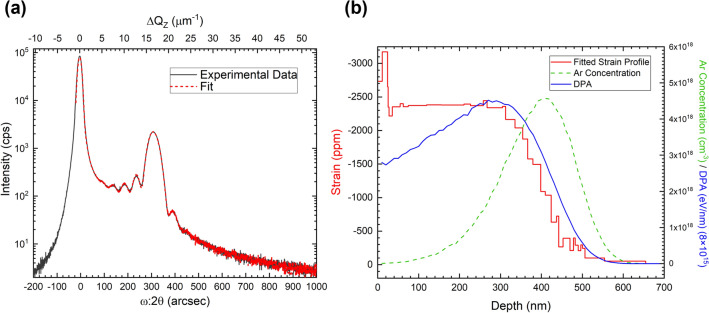
(**a**) Experimental ω:2θ scan of the (3030) reflection versus (**b**) simulated strain model and SRIM Ar^+^ concentration and displacements-per-atom (DPA) profiles. The substrate peak at ω:2θ = 0″ (ΔQ_Z_ = 0 μm^−1^) corresponds to a distance of 6945 μm^−1^ along Q_Z_ from the reciprocal space origin. The strain peak at ~ 310″ corresponds to a distance of 6957 μm^−1^ along Q_Z_ from the reciprocal space origin.

The symmetric ω:2θ line scan of the (3030) reflection is shown in Fig. 1a. The intense peak at the origin is due to the underlying, unstrained material beneath the implanted region. The peak at ~ 310″ and oscillations to the right of the main substrate peak correspond to compressive strain induced by the Ar^+^ implantation. For most materials^7–12^, the strain peak and oscillations due implantation appears on the left side of the main substrate peak (i.e. tensile strain)^7^ Note that this symmetric ω:2θ scan measures the strain along the sample surface normal. Dynamical diffraction simulations (RADS)^15^ were employed to quantify the strain distribution as shown in Fig. 1b along with the Ar^+^ concentration and displacements-per-atom profile calculated using SRIM^16^. We observe that the resulting compressive strain profile generally follows the displacements-per-atom profile calculated from SRIM, and this is similar to what is observed with other implanted materials^7,17,18^. We find that different parts of the resulting strain model corresponds to different sections of the XRD ω:2θ measurement: (1) the shallow highly strained surface layer (~ 30 nm from the surface) corresponds to the rightmost fringe at ~ 400″ in the experimental XRD measurement, (2) the constant strain region that spans from ~ 30 nm to ~ 310 nm from the surface corresponds to the strain peak at ~ 310″, and (3) tail of the strain profile spanning ~ 310 nm from the surface to the bulk of the substrate past the implanted region corresponds to the fringes to the left of the strain peak (~ 100″ to ~ 250″). We note however, that because this an interference effect, each feature can have contributions from different depths but overall these associations hold for this set of samples. We also note that the implant conditions are such that the strain generated due to displaced atoms dominates over the chemical contributions of the Ar concentration (Ar concentration is only ~ 0.005 at%).

**Figure 2:**
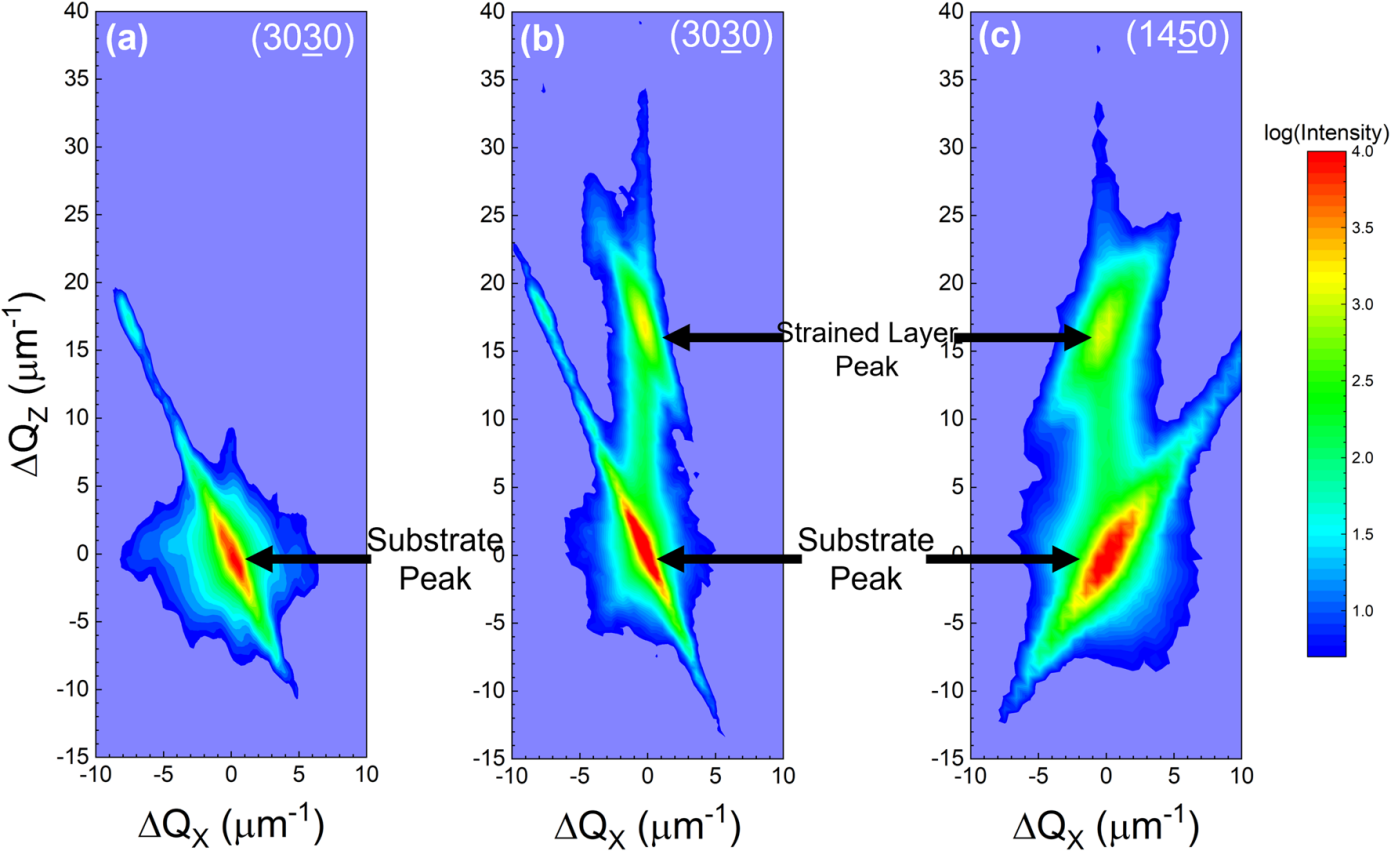
Reciprocal space maps for the (**a**) symmetric 3030 reflection prior to implantation, (**b**) symmetric 3030 reflection after implantation, and (**c**) asymmetric 1450 reflection after implantation. The upper peaks correspond to the strained implanted calcite layer while the lower peaks correspond to the substrate. Q_X_ and Q_Z_ correspond to the [1210]* and [1010]* directions, respectively. The non-vertical streaks observed at both the substrate and strained layer peaks are due to the incident and scattered beam optical elements of the X-ray diffractometer.

**Figure 3:**
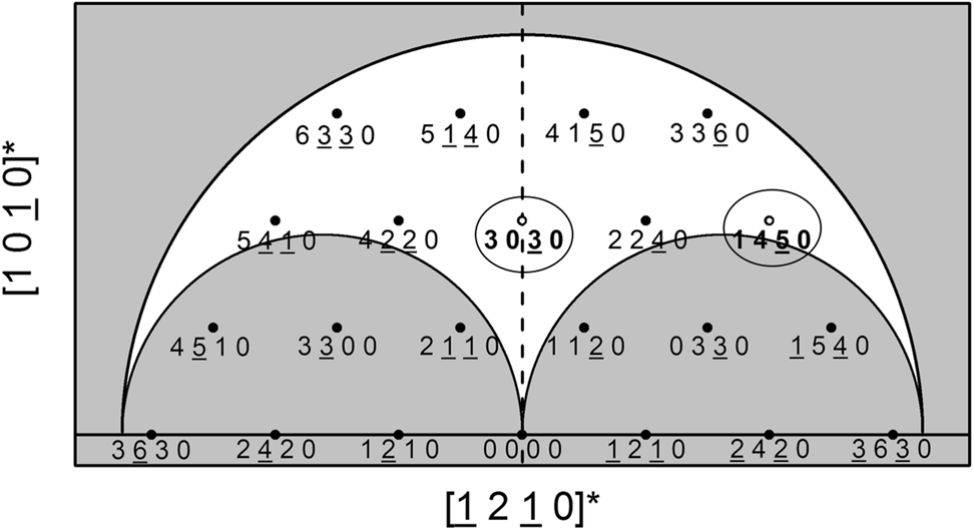
Cross-section of reciprocal space spanned by the [1010]* and [1210]* zone axes for the allowed reflections of calcite. The open circles indicate peaks measured in this study. The outer shaded region corresponds to the Cu Kα_1_ limiting sphere and the two inner shaded regions correspond to transmission geometry conditions.

In order to further assess the strain state of the implanted calcite, reciprocal space maps (RSMs) of the symmetric reciprocal lattice point 3030 and asymmetric reciprocal lattice point 1450 were generated as shown in Fig. 2. The origin (Q_x_, Q_z_ = 0) corresponds to the unstrained substrate peak which is a distance of 6945 μm^−1^ from the reciprocal space origin. While the symmetric 3030 RSM contains only out-of-plane strain information (see Fig. 1a, which corresponds to a single vertical line scan through the centers of the peaks in Fig. 2a), the asymmetric 1450 reciprocal lattice point contains both in-plane and out-of-plane strain information. The 1450 RSM shows that both the main substrate and strained layer points are aligned along the ordinate axis Q_Z_ (both points share the same Q_z_ value and once again, the origin is at the unstrained substrate peak position (which is 8020 μm^−1^ away from the reciprocal space origin along Q_x_). This match of the Q_z_ values and for both reflections and the fact that the (1450) unstrained peak and strained peak share the same Q_x_ value confirms that the strained layer is pseudomorphically strained along the in-plane directions with the underlying unstrained bulk substrate below the implanted region. Thus, while the implanted region is experiencing in-plane biaxial compressive stress from the underlying substrate, the implanted region also exhibits out-of-plane lattice compression—a characteristic of an auxetic material. This behavior is highly atypical for ion-implantation induced strain, with the usual case being that the in-plane compression produces an out-of-plane expansion. Furthermore, the vertical distance between the main substrate and strain layer points is the same between the symmetric 3030 and asymmetric 1450 reciprocal lattice points as shown in Fig. 2. This is expected because these two reflections share the same out-of-plane component along Q_Z_ away from the origin in reciprocal space as shown in the slice of reciprocal space in Fig. 3. The RSMs provide another visual representation of the implant-induced strain. Points farther away from the origin in reciprocal space correspond to smaller real space dimensions. Thus, a layer exhibiting compressive strain corresponds to reciprocal lattice points further away from the reciprocal space origin.

**Figure 4:**
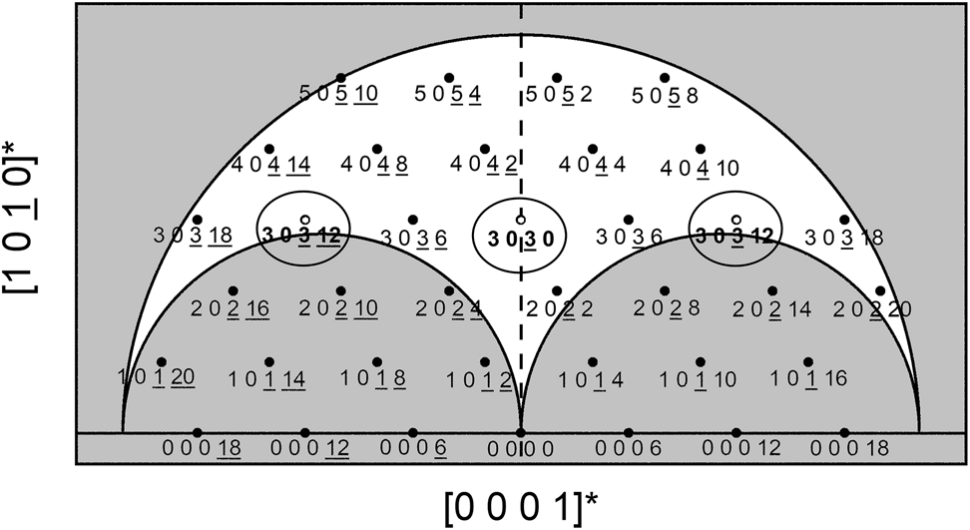
Cross-section of reciprocal space spanned by the [1010]* and [0001]* zone axes for the allowed reflections of calcite. The open circles indicate peaks measured in this study. The outer shaded region corresponds to the Cu Kα_1_ limiting sphere and the two inner shaded regions correspond to transmission geometry conditions.

**Figure 5:**
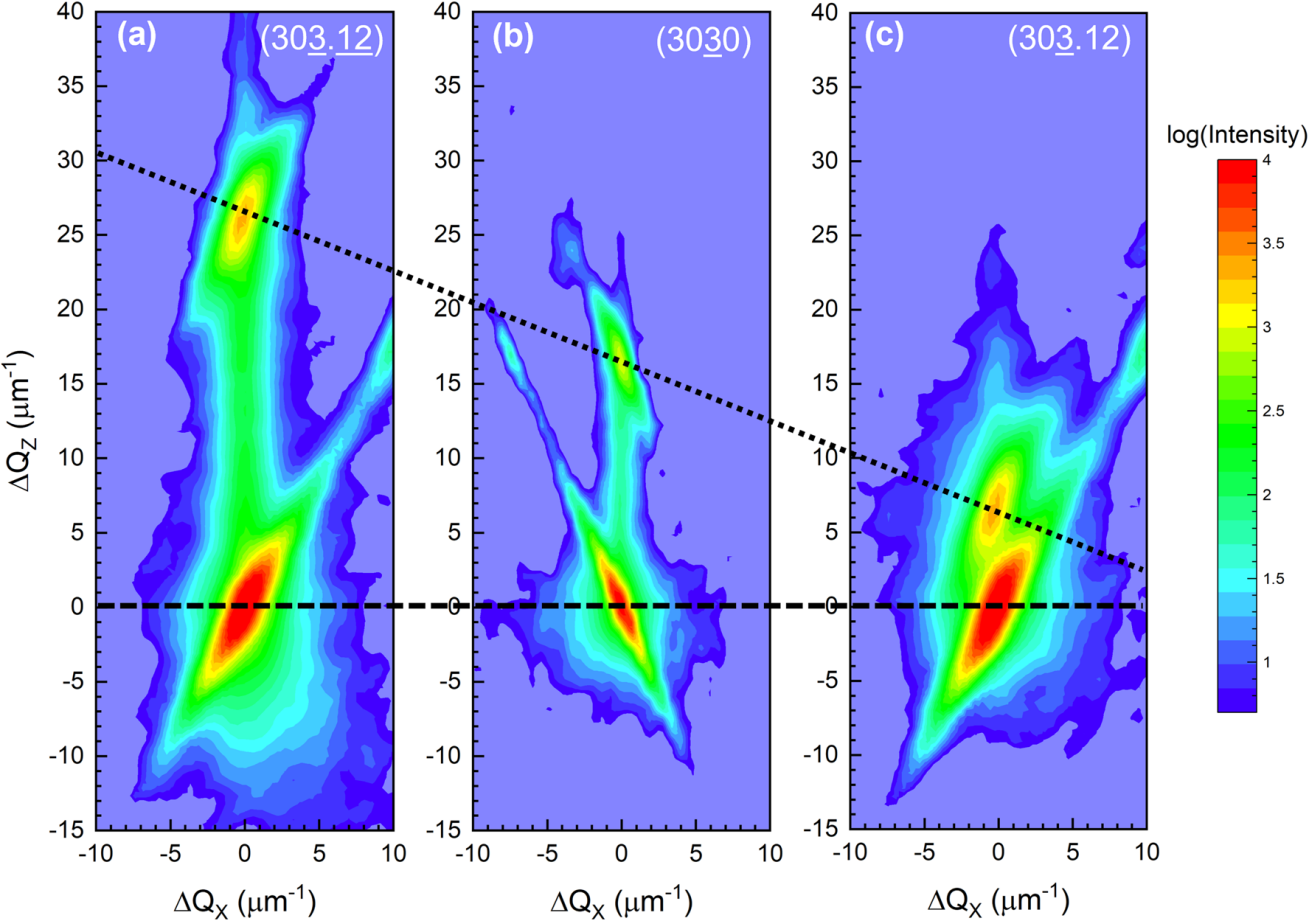
Reciprocal space maps for the (**a**) asymmetric 303.12 (**b**) symmetric 3030, and (**c**) asymmetric 303.12 reflection. The upper peaks in each RSM correspond to the strained implanted calcite layer while the lower peaks correspond to the substrate. Q_X_ and Q_Z_ correspond to the [0001]* and [1010]* directions, respectively. The horizontal dashed line corresponds to the substrate peaks, while the diagonal dotted line corresponds to the strained layer peaks. The non-vertical streaks observed at both the substrate and strained layer peaks are due to the incident and scattered beam optical elements of the X-ray diffractometer. Both 303.12 and 303.12 RSMs were measured in the glancing exit geometry, where 303.12 was measured such that the sample was rotated in-plane 180° with respect to the 3030 and 303.12 RSMs^28^.

RSMs were also generated for the 303.12, 3030, and 303.12 reflections by rotating the sample in-plane by 90° (i.e. aligning the samples such that the [0001] calcite zone axis was parallel to the incident X-ray beam). The slice of reciprocal space for the zone axis containing these reflections is shown in Fig. 4. Analogous to the 1450 reflection, the asymmetric 303.12 and 303.12 reflections have the same out-of-plane component along Q_Z_ as the symmetric 3030 reflection. However, while the measured RSMs for the 303.12, 3030, and 303.12 reflections in Fig. 5 show the strained layer is pseudomorphically strained, these RSMs also reveal that the strained layer exhibits shearing of the unit cell along the in-plane [0001] axis. Even though these reflections all share the same component along [1010], the reciprocal lattice points of the 303.12, 3030, and 303.12 reflections for the strained layer peak do not form a line parallel to the horizontal along Q_X_ as shown in Fig. 5, unlike what was observed for the 3030 and 1450 RSMs in Fig. 2 (and is typically observed for strained layers). The angle of inclination corresponds to the shear angle^19^, which is found to be only ~ 0.08° along the [0001] c-axis. Note that in Fig. 5 the RSMs for each reflection were measured separately, and the 303.12 (Fig. 5a) and 303.12 (Fig. 5c) RSMs are separated by d_000.12_ = 7037 μm^−1^ along the Q_X_ axis from the 3030 RSM (Fig. 5b). The origin of this shear is likely due to the miscut of the calcite substrate, i.e. the true surface plane of the (1010) calcite used in this study is more accurately represented by a high-index plane^20^. The miscut was measured to be ~ 1° towards an in-plane direction ~ 17° away from the [0001]. De Caro et al.^21^ demonstrated that the biaxial stress applied by substrates with surface orientations that lack at least two-fold symmetry will induce shear on heteroepitaxial layers. The implanted region can be thought of as a heterolayer for our study because the implanted layer experiences in-plane biaxial stress. The surface plane of the vicinal (1010) calcite substrates with a 1° miscut lacks even two-fold symmetry. Therefore, shear is not expected to occur if on-axis (1010) calcite substrates were implanted. Furthermore, De Caro et al.^21^ concluded that the direction of shear will occur along the in-plane direction that exhibits the highest symmetry, which is [0001] for (1010) calcite substrates—consistent with our experimental observations. Molecular dynamics simulations suggest either a decrease^13^ in density for Ar^+^ ion energies > 1 keV or no change^14^ in density for an ion energy ~ 400 keV. Another study claims upon neutron irradiation of calcite the density decreases without describing how the measurement was performed^22^. In our studies, the unstrained material has a density of 2.711 g/cm^3^ (using the in-plane d_1210_ = 4010 μm^−1^ and d_0001_ = 586 μm^−1^, and the out-of plane d_1010_ = 2315 μm^−1^); the density of the strained layer is ~ 0.2% greater. The unit cell angles for the unstrained substrate are α = β = 90° and γ = 120° where α is the angle between the b- and c-axes, β is the angle between the a- and c-axes, and γ is the angle between the a- and b-axes. The strained epitaxial layer possesses the same d_1210_ and d_0001_ values, but a d_1010_ = 2319 μm^−1^ and α = 90°, β = 89.93° (the β angle spans only a partial component along the measured shearing direction), and γ = 120°. Thus, the implanted calcite density is increased within the implanted region primarily due to the out-of-plane lattice contraction. In all of the RSMs, the non-vertical streaks observed are associated with the incident and scattered beam optical elements.

Based on the mechanism for auxeticity in α-cristobalite and α-quartz, which involves the rotations of corner-sharing SiO_4_ tetrahedrals^3,23,24^, we speculate that the mechanism for auxeticity in calcite may be similarly attributed to the rotations of the carbonate groups that occupy the corners of the corner-sharing octahedrals in calcite. Metastable phase transformations (calcite II–V) of calcite have been observed when subjected to high pressures^25–27^. In response to pressure, the carbonate groups rotate, the corner-sharing octahedrals distort, and the density increases^25,27^, in agreement with the increased density observed in the X-ray scattering measurements. In this current work, ion implanting into the vicinal (1010)-oriented trigonal substrates results in a triclinic distortion. An auxetic material, however, will respond by exhibiting an increase in density post-implantation, whereas a non-auxetic material will exhibit a decrease in density.

In this study, auxeticity in calcite is experimentally verified for the first time—a property in calcite that had only been theoretically predicted^6^. While ion implantation has been previously used for other applications such as exfoliation and doping^7,9–12,17^, we employ ion implantation along with X-ray diffraction measurements to probe the mechanical properties of calcite. Fundamental understanding of the mechanical properties can be insightful for studies examining, for example, the effects of irradiation on the various constituents of concrete (e.g. calcite)^13,14^. Furthermore, both the magnitude and type (tensile vs compressive) strain have been known to influence other materials characteristics such as electronic^29,30^ and thermal transport^31,32^.

## Materials and methods

### Sample preparation

Polished 1-mm thick (1010) oriented single crystalline calcite substrates were sourced from MTI Corporation. The calcite substrates were then implanted at room temperature without active cooling with Ar^+^ with an energy of 400 keV and dose of 1 × 10^14^ cm^−2^ at the Michigan Ion Beam Laboratory. Further implantation with active cooling is underway.

### Triple-axis X-ray diffraction and modeling

Both the symmetric ω:2θ line scan of the (3030) reflection as well as symmetric 3030 and asymmetric 1450 and 303.12 reciprocal space maps were measured using a high-resolution Bruker-JV D1 diffractometer. The conditioning for the incident beam includes a Göbel mirror^33^ and a (220) channel-cut silicon crystal, which results in a highly collimated monochromatic beam of Cu Kα_1_ radiation. For the (3030) symmetric reflection measurements, the scattered beam optics used is a 4-bounce (220) channel-cut silicon crystal. For all the asymmetric reflections, the optics used are ~ 0.14 mm narrow slits with an acceptance angle of ~ 100″. The lattice parameter of calcite along the a-axis is 0.49877 nm^27^, which corresponds to a (3030) Bragg angle of 32.3°. The Bruker RADS^15^ software was employed to simulate and obtain a strain profile by modeling the symmetric ω:2θ line scan. This software utilizes a genetic algorithm called “Differential Evolution^34^.” The input parameters were modified to extract strain information from a trigonal unit cell.

## Data Availability

The data that support the findings of this study are available from the corresponding author upon reasonable request.
